# Post-transplant ureteral stenosis: donor-related risk factors, graft outcome and surgical management

**DOI:** 10.3389/fneph.2026.1943229

**Published:** 2026-09-24

**Authors:** T. Wagner, Hauke Heinrich, E. Schober, L. Ischebeck, N. Schleussner, M. Hummels, F. C. Koehler, C. Kurschat, M. Kann, C. Bruns, D. L. Stippel, M. N. Thomas

**Affiliations:** 1 Department of General, Visceral, Thoracic and Transplant Surgery, University of Cologne, Faculty of Medicine and University Hospital Cologne, Cologne, Germany; 2 Department of Surgery, Beth Israel Deaconess Medical Center, Harvard University, Boston, MA, United States; 3 Max-Planck-Institute for Biology of Ageing, University of Cologne, Faculty of Medicine and University Hospital Cologne, Cologne, Germany; 4 Department II of Internal Medicine and Center for Molecular Medicine Cologne, University of Cologne, Faculty of Medicine and University Hospital Cologne, Cologne, Germany; 5 Cologne Excellence Cluster on Cellular Stress Responses in Aging-Associated Diseases (CECAD), Faculty of Medicine, University of Cologne, Cologne, Germany; 6 Department of Internal Medicine (Nephrology) & Einthoven Laboratory of Vascular and Regenerative Medicine, Leiden University Medical Center, Leiden, Netherlands

**Keywords:** graft survival, kidney transplantation, surgical revision, ureteral stenosis, ureteral transposition, ureteroneocystostomy

## Abstract

**Introduction:**

Kidney transplantation remains the preferred treatment for end-stage renal disease; however, the increasing use of extended-criteria donors has been associated with a higher incidence of postoperative complications.

**Methods:**

We retrospectively analyzed 471 kidney transplant recipients at the University of Cologne between 2012 and 2022 to evaluate the incidence, risk factors, clinical outcomes, and surgical management of ureteral stenosis.

**Results:**

Ureteral stenosis occurred in 6.37% and was significantly associated with older donor age within the extended-criteria donor range (65 vs. 56 years, p = 0.006), multiple renal arteries (33.3% vs. 10.2%, p < 0.001), and BK virus infection, as reflected by higher rates of BK viruria (26.7% vs. 7.0%, p = 0.002) and BK viraemia (26.7% vs. 8.6%, p = 0.005). Renal function was significantly impaired in recipients with ureteral stenosis at 6 months, 1 year, and 2 years after transplantation. Death-censored graft survival was significantly lower in the stenosis group (79.7% vs. 93.3%, p = 0.01).

**Discussion:**

Ureteral transposition and ureteroneocystostomy demonstrated comparable outcomes, long-term renal function and graft survival supporting individualization of surgical technique based on anatomical and patient-specific factors.

## Introduction

Kidney transplantation is the treatment of choice in end-stage renal disease, improving the quality of life and life expectancy (1–3). Due to the increasing demand for kidney transplants and organ shortage, the number of extended criteria donors (ECD) is increasing (4–7). However, ECD transplants are associated with a higher risk of postoperative complications (8). Ureteral stenosis is the most common urological complication after kidney transplantation, with a reported incidence of 0.5% to over 20%, classified as early or late onset (9–11). Early strictures, occurring within the first three months post-transplant, are typically attributed to surgical factors, ischemic injury or periureteral fibrosis (9, 12, 13). In contrast, late strictures are linked to viral reactivation, particularly BK virus (BKV) (14, 15). While BKV is a latent polyomavirus present in up to 90% of the adult population, its reactivation under immunosuppression has been associated with nephropathy and ureteral obstruction. However, its causal role in stricture formation remains debated (16, 17). Risk factors have been identified, including advanced donor age (>65 years), male recipient sex, multiple renal arteries, delayed graft function, prolonged cold ischemia time, and postoperative fluid collections (9, 18–20). The two most widely applied techniques for the initial ureteral reconstruction (ureteroneocystostomy) in kidney transplantation are Politano-Leadbetter (PL) and Lich-Gregoir (LG) (21). A meta-analysis by Alberts et al. further demonstrated lower rates of urinary leakage and hematuria with LG, while postoperative rates of ureteral stenosis did not differ significantly between the two techniques (22). Therefore, the current European Association of Urology guideline recommends LG as the preferred method for kidney transplantation (13, 22). Nevertheless, the influence of surgical technique remains controversial, with conflicting evidence that may relate more to refluxing versus non-refluxing approaches than to ureteral complications such as stenosis (13, 23). Surgical interventions for ureteral stenosis following renal transplantation encompass a range of therapeutic strategies, with the selection of an appropriate approach contingent upon the anatomical location and extent of the stenosis, as well as the interval elapsed between transplantation and diagnosis (24, 25). Established techniques include ureteroneocystostomy and pyeloureterostomy, among which the extravesical ureteroneocystostomy according to the Lich–Gregoir technique has emerged as the most widely adopted (10, 11, 26). In cases involving long-segment strictures or proximal and mid-ureteral lesions, ureteroureterostomy or pyeloureterocystostomy may represent viable alternatives, provided the integrity of the native ureter is confirmed (10). Given their capacity to compromise graft function, augment morbidity, and necessitate repeated interventions, ureteral strictures constitute a significant complication in the post-transplant setting. The present retrospective single-center study aims to evaluate their incidence, delineate associated risk factors, and assess the outcome of differing treatment strategies on long-term graft and patient survival.

## Materials and methods

This study was reviewed and approved by the Ethics Committee of the Medical Faculty of the University of Cologne (ethical approval number 25-1012-retro). Authors declare that organs were procured by the Department of General, Visceral, Thoracic and Transplant Surgery, University Hospital Cologne, and sourced ethically in accordance with German transplantation law (Transplantationsgesetz, TPG) and the principles of the Declaration of Istanbul and the WHO Guiding Principles on Human Cell, Tissue and Organ Transplantation. Renal transplantations at our institution between January 2012 and December 2022 were systematically screened and individually reviewed for eligibility. As ureteral complications typically manifest within the postoperative follow-up period, a study inclusion cutoff of December 2022 was applied to ensure adequate observational time for the detection and documentation of ureteral stenosis in all included patients. Patients below 18 years of age at the time of transplantation and those undergoing combined organ transplant procedures were excluded from analysis. Patient selection and eligibility are reported in accordance with CONSORT guidelines and presented as a flow diagram (**Figure 1**). Renal transplantation was performed using a standardized extraperitoneal iliac fossa approach. Following preparation of the retroperitoneal operative bed, vascular anastomoses were established to the external iliac vessels (11, 27). Urinary tract reconstruction was performed either by means of an extravesical ureteroneocystostomy according to the modified Lich–Gregoir technique (LG) or an intravesical ureteroneocystostomy using the Politano–Leadbetter technique (PL) (11, 21, 27). In all procedures, an anti-reflux mechanism was created, and a double-J ureteral stent was routinely inserted with scheduled removal after 30 days (9, 28).

**Figure 1:**
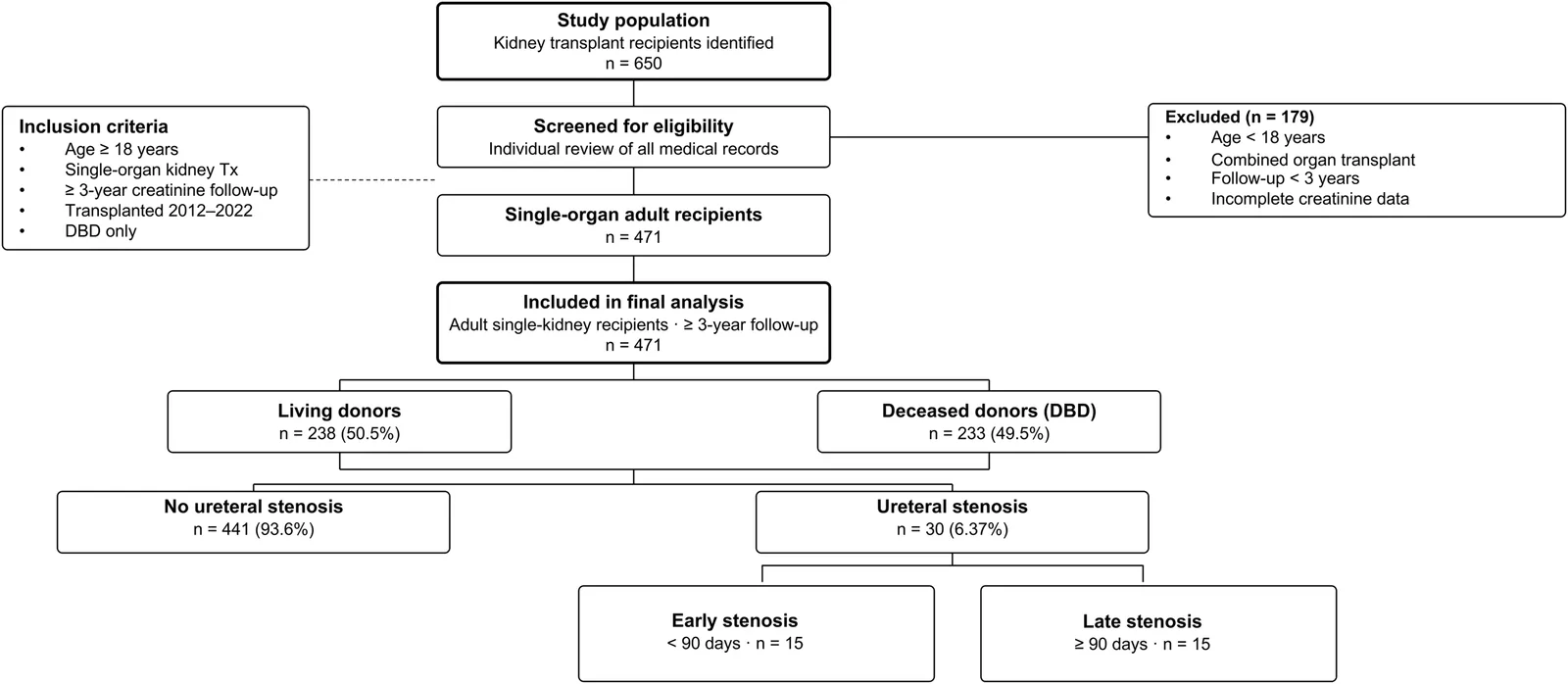
Consort flow diagram depicting participant selection from 650 kidney transplant recipients identified at the University Hospital Cologne between January 2012 and December 2022. After exclusion of pediatric recipients, combined organ transplants, and cases with less than three years of follow-up, 471 adult single-kidney transplant recipients were included in the final analysis. Of these, 30 patients (6.37%) developed ureteral stenosis and were further stratified into early-onset (< 90 days, n = 15) and late-onset (≥ 90 days, n = 15) subgroups.

All recipients received standardized immunosuppressive therapy consisting of a calcineurin inhibitor, mycophenolate mofetil, and corticosteroids; induction immunosuppression was administered according to individual immunological risk stratification based on the virtual panel-reactive antibody (vPRA) level, with basiliximab administered to low-risk recipients and thymoglobulin reserved for those at high immunological risk (29). Antimicrobial prophylaxis included trimethoprim-sulfamethoxazole for pneumocystis jirovecii pneumonia (PCP) and valganciclovir-based prophylaxis for cytomegalovirus (CMV), stratified by donor and recipient serostatus; in high-risk populations, specifically CMV seropositive recipients (R+) receiving induction immunosuppression, prophylactic (val)ganciclovir was administered for a minimum duration of three months post-transplantation (30–33). Study data were collected regarding clinical and surgical characteristics of both donor and recipient, such as demographics, comorbidities, transplant history, vascular anatomy and ischemia times. In accordance with national quality standards (G-BA, QSKH-RL), which mandate annual follow-up visits for at least three years following living donor kidney transplantation, follow-up data were available for the living donor recipients; however, deceased donor recipients were partly followed at external centers, resulting in a loss of follow-up (34). BKV replication was quantified in urine and blood by nucleic acid testing and reported as copies/mL. Plasma BKV-DNA was assessed as part of routine post-transplant surveillance, whereas urinary BKV loads were determined on clinical indication. High-level viruria was defined as >10^7^ copies/mL, while plasma BKV-DNA >10^4^ copies/mL was considered clinically significant (35). Viral loads were reported as median and IQR. All medical records and pathology reports were reviewed to ascertain the presence or absence of ureteral complications. Diagnosis of ureteral stenosis was defined as the presence of hydronephrosis with dilatation of the renal calyces and pelvis on ultrasound, combined with impaired graft function as reflected by rising serum creatinine levels, after exclusion of other causes of graft dysfunction including acute rejection, postoperative hematoma, lymphocele and urinary collections; diagnosis was confirmed by antegrade or retrograde pyelography if clinically indicated (9, 19). Ureteral stenoses were categorized according to the time of onset as early (<90 days post-transplant) or late (≥90 days post-transplant). The anatomical location of the stenosis and the management strategy (surgical revision or conservative treatment) were documented. Treatment was individualized according to the severity and persistence of obstruction and patient-specific operative risk, with a general institutional preference for definitive surgical management. The choice of surgical technique was made by two experienced transplant surgeons based on stenosis location, the extent of viable transplant ureter, and the suitability of the native ureter for reconstruction.

The applied techniques were ureteroneocystostomy, pyeloureterostomy and ureteroureterostomy (see **Supplementary Figure 1**). In ureteroneocystostomy the donor ureter is implanted directly into the recipient bladder, most commonly by the extravesical Lich-Gregoir technique, in which the detrusor is incised, the spatulated ureter is anastomosed to the exposed bladder mucosa and the muscle layer is closed over it to create an antireflux tunnel (11). In the transposition procedures the urinary tract is instead restored to the ipsilateral native ureter either from the graft renal pelvis (pyeloureterostomy) or from the graft ureter (ureteroureterostomy), usually as an end-to-end anastomosis to the spatulated distal native ureter after simple proximal ligation of the native ureter with the native kidney left *in situ*, or alternatively end-to-side leaving the native ureter in continuity, in all cases combined with a double-J stent implantation (11, 36, 37). Ureteroureterostomy and pyeloureterostomy were grouped together as ‘transposition’ (anastomosis to the native ureter) and compared to ureteroneocystostomy with respect to perioperative parameters and long-term creatinine- based outcome. The primary outcome in the initial cohort analysis was the incidence of ureteral stenosis. Secondary outcomes included overall graft survival, patient survival, renal function and direct comparison of the revision techniques. Serum creatinine levels were systematically collected before and after the diagnosis of ureteral stenosis.

### Statistical analysis

For descriptive statistics patients were stratified into “Ureter stenosis” and “No ureteral stenosis”. For the surgical management of ureteral stenosis groups were stratified into “transposition” and “neocystostomy”. Continuous variables were expressed as median with the interquartile range in parentheses, while categorial variables were expressed as counts with percentage in parentheses. Statistical tests used to compare the groups were Wilcoxon-rank-sum test (continuous), Pearson’s Chi-squared test (binary), or Fisher’s exact test (categorial). For creatinine follow up, Mann-Whitney-U test with Bonferroni correction was used for statistical analysis. For graft-survival, patient survival and death-censored graft survival, we performed log-rank tests for statistical analysis. 95% confidence intervals were calculated using Greenwood variance with log-log transformation. For all analyses, a p-value < 0.05 was defined as statistically significant. All statistical analyses and figures were performed and generated using Python (version 3.11.9; Python Software Foundation, Wilmington, DE, USA).

## Results

Between January 2012 and December 2022, 650 kidney transplantations were performed. After excluding pediatric recipients, combined organ transplants and cases with less than 3 years of follow-up, 471 adult single-kidney transplant recipients were included in the final analysis. 238 (50.5%) grafts were from living donors and 233 (49.5%) from deceased donors.

### Recipient characteristics

No significant differences in recipient demographic characteristics were observed between the non-stenosis and stenosis group (**Table 1**). Median recipient age was 53 years (IQR 39.0–62.0) in the non-stenosis group and 53 years (IQR 34.0–64.8) in the stenosis group (p = 0.88). Sex distribution was likewise comparable, with female patients accounting for 37.6% and 36.7% of the non-stenosis and stenosis groups, respectively (p > 0.9). Median BMI was similar between groups, at 26.3 kg/m² (IQR 23.1–30.1) and 25.5 kg/m² (IQR 23.2–28.2), respectively (p = 0.462). Implantation side showed no significant difference, with left-sided implantation performed in 41.8% vs. 40.0% of cases (p > 0.9). Initial ureteral reconstruction was documented in all 471 patients. The Politano–Leadbetter technique was used in 432 patients (91.7%) and the Lich–Gregoir technique in 33 patients (7.0%), while 6 patients (1.3%) underwent alternative urinary reconstruction (including anastomosis to a neobladder (n = 2), transcutaneous urinary diversion (n = 3), and ileal conduit reconstruction (n = 1). The overall distribution of reconstruction techniques was comparable between groups (p = 0.471). In the direct comparison of the two standard techniques, ureteral stenosis occurred in 30 of 432 patients (6.9%) after Politano–Leadbetter reconstruction and in none of the 33 patients (0.0%) after Lich–Gregoir reconstruction; however, this difference did not reach statistical significance (Fisher’s exact test, p = 0.154).

**Table 1:** Recipient characteristics.

Characteristic	N	Overall	No stenosis	Stenosis	p-value²
		N = 471¹	N = 441¹	N = 30¹	
Recipient age [years]	471	53 (39, 62)	53 (39, 62)	53 (34, 64.8)	0.88
*(Missing)*		*0*	*0*	*0*	
Recipient Sex	471				>0.9
Female		177 (37.6%)	166 (37.6%)	11 (36.7%)	
Male		294 (62.4%)	275 (62.4%)	19 (63.3%)	
*(Missing)*		*0*	*0*	*0*	
Weight [kg]	467	80 (66.7, 90)	80 (66, 92)	81 (70, 85.8)	0.726
*(Missing)*		*4*	*4*	*0*	
Height [m]	467	1.7 (1.6, 1.8)	1.7 (1.6, 1.8)	1.8 (1.7, 1.8)	0.472
*(Missing)*		*4*	*4*	*0*	
BMI [kg/m²]	467	26.3 (23.0, 29.8)	26.3 (23.1, 30.1)	25.5 (23.2, 28.2)	0.462
*(Missing)*		*4*	*4*	*0*	
Disease group	470				>0.9
Atrophic Kidneys		12 (2.6%)	12 (2.7%)	0 (0.0%)	
Cystic Kidney Diseases		98 (20.9%)	90 (20.5%)	8 (26.7%)	
Diabetic Nephropathy		58 (12.3%)	54 (12.3%)	4 (13.3%)	
Glomerular Diseases		191 (40.6%)	182 (41.4%)	9 (30.0%)	
Hypertensive/Vascular Nephropathy		37 (7.9%)	33 (7.5%)	4 (13.3%)	
Infectious/post-infectious		5 (1.1%)	5 (1.1%)	0 (0.0%)	
Neoplastic		0 (0.0%)	0 (0.0%)	0 (0.0%)	
Others		2 (0.4%)	2 (0.5%)	0 (0.0%)	
Systemic Diseases Affecting Kidneys		12 (2.6%)	11 (2.5%)	1 (3.3%)	
Transplant-Related		3 (0.6%)	3 (0.7%)	0 (0.0%)	
Traumatic/Iatrogenic		2 (0.4%)	2 (0.5%)	0 (0.0%)	
Tubulointerstitial Diseases		6 (1.3%)	6 (1.4%)	0 (0.0%)	
Congenital/Hereditary Kidney Diseases		22 (4.7%)	20 (4.5%)	2 (6.7%)	
Unknown		22 (4.7%)	20 (4.5%)	2 (6.7%)	
*(Missing)*		*1*	*1*	*0*	
Preemptive transplant	471				0.639
yes		119 (25.3%)	113 (25.6%)	6 (20.0%)	
no		352 (74.7%)	328 (74.4%)	24 (80.0%)	
*(Missing)*		*0*	*0*	*0*	
Implantation side	470				>0.9
Left		196 (41.7%)	184 (41.8%)	12 (40.0%)	
Right		274 (58.3%)	256 (58.2%)	18 (60.0%)	
Initial reconstruction of ureter	471				0.471
Lich-Gregoir		33 (7.0%)	33 (7.5%)	0 (0.0%)	
Politano-Leadbetter		432 (91.7%)	402 (91.2%)	30 (100.0%)	
Other		6 (1.3%)	6 (1.4%)	0 (0.0%)	
*(Missing)*		*0*	*0*	*0*	

²Wilcoxon rank sum test; Pearson's Chi-squared test; Fisher's exact test.

### Donor characteristics

In contrast to recipient characteristics, donor-related variables showed significant differences between groups (**Table 2**). Donors in the stenosis group were significantly older, with a median age of 65 years (IQR 57.0–71.4) compared to 56 years (IQR 48–65) in the non-stenosis group (p = 0.006) and of shorter stature (1.70 vs. 1.73 m, p = 0.016). Donation type (living vs. deceased) was comparable between groups (50.5% vs. 50.0%, p > 0.9), as was donor sex, with a proportion of female donors of 47.9% vs. 65.5% (p = 0.1). The presence of multiple renal arteries was significantly more frequent in the stenosis group (p < 0.001). Donor kidney side was similarly distributed, with a proportion of left-sided kidneys of 59.3% vs. 50.0% (p = 0.417). ECD status was assessed separately for deceased and living donors and stratified by stenosis onset (**Supplementary Tables S1**–**3**). Among deceased donors, the proportion of donors aged ≥60 years was numerically higher in the early stenosis group (100% vs. 50.0%, p = 0.085), and multiple renal arteries were equally distributed between groups (42.9% vs. 37.5%, p > 0.9). No patient exceeded a total ischemia time of 24 hours. Among living donors, ECD-related criteria were likewise comparable between early and late stenosis groups.

**Table 2:** Donor characteristics.

Characteristic	N	Overall	No stenosis	Stenosis	p-value²
		N = 471¹	N = 441¹	N = 30¹	
Living donation	471	238 (50.5%)	223 (50.5%)	15 (50.0%)	>0.9
*(Missing)*		*0*	*0*	*0*	
*Age [years]*	*461*	*57 (48, 66)*	*56 (48, 65)*	*65 (57, 71.4)*	*0.006*
*(Missing)*		*10*	*9*	*1*	
Sex	461				0.1
Female		226 (49.0%)	207 (47.9%)	19 (65.5%)	
Male		235 (51.0%)	225 (52.1%)	10 (34.5%)	
*(Missing)*		*10*	*9*	*1*	
Weight [kg]	461	80 (70, 90)	80 (70, 90)	75 (70, 89)	0.47
*(Missing)*		*10*	*9*	*1*	
Height [m]	461	1.7 (1.6, 1.8)	1.7 (1.6, 1.8)	1.7 (1.6, 1.7)	0.016
(Missing)		10	9	1	
BMI [kg/m²]	461	26.1 (23.9, 29.6)	26.1 (23.8, 29.5)	26.2 (24.5, 30.8)	0.368
*(Missing)*		*10*	*9*	*1*	
Blood group	455				0.212
A		190 (41.8%)	180 (42.1%)	10 (37.0%)	
B		48 (10.5%)	47 (11.0%)	1 (3.7%)	
AB		21 (4.6%)	21 (4.9%)	0 (0.0%)	
O		196 (43.1%)	180 (42.1%)	16 (59.3%)	
*(Missing)*		*16*	*13*	*3*	
Number of arteries	470				<0.001
1.0		415 (88.3%)	395 (89.8%)	20 (66.7%)	
2.0		49 (10.4%)	42 (9.5%)	7 (23.3%)	
3.0		5 (1.1%)	2 (0.5%)	3 (10.0%)	
4.0		1 (0.2%)	1 (0.2%)	0 (0.0%)	
*(Missing)*		*1*	*1*	*0*	
Kidney side	470				0.417
left		276 (58.7%)	261 (59.3%)	15 (50.0%)	
right		194 (41.3%)	179 (40.7%)	15 (50.0%)	
*(Missing)*		*1*	*1*	*0*	

¹n (%); Median (Q1, Q3)

²Pearson's Chi-squared test; Wilcoxon rank sum test; Fisher's exact test.

### Perioperative parameters

Perioperative parameters were comparable between groups (**Table 3**). Median operative time was comparable between groups (154 vs. 158.5 minutes, p = 0.757). Delayed graft function occurred in 6.6% of the non-stenosis group and in none of the stenosis group (p = 0.243), and primary non-function was similarly distributed (4.5% vs. 0%, p = 0.629). Median cold ischemic time (238 min, IQR 173.0–578.5 vs. 230.5 min, IQR 182.0–649.0, p = 0.757) and median warm ischemic time (35 min, IQR 30–49 vs. 34 min, IQR 29–43.8, p = 0.302) were likewise comparable between groups. Overall postoperative complication rates did not differ significantly between groups (p = 0.587). According to the Clavien-Dindo Classification, the majority of patients in both groups experienced no complications (47.4% vs. 53.3%). The distribution across complication grades is detailed in **Table 3**. Notably, no patient in the stenosis group experienced multi-organ dysfunction or mortality attributable to surgery. Lymphocele incidence was numerically higher in the stenosis group, narrowly failed to reach statistical significance (8.8% vs. 20.0%, p = 0.055). Postoperative hematoma (6.3% vs. 3.3%, p > 0.9), wound infection (5.2% vs. 0%, p = 0.386), myocardial infarction (4.1% vs. 0%, p = 0.619), and pulmonary embolism (1.8% vs. 3.3%, p = 0.45) were all comparable between groups.

**Table 3:** Perioperative characteristics.

Characteristic	N	Overall	No Stenosis	Stenosis	p-value²
		N = 471¹	N = 441¹	N = 30¹	
Primary Non-Function (PNF)	471	20 (4.2%)	20 (4.5%)	0 (0.0%)	0.629
*(Missing)*		*0*	*0*	*0*	
Delayed Graft Function (DGF)	471	29 (6.2%)	29 (6.6%)	0 (0.0%)	0.243
*(Missing)*		*0*	*0*	*0*	
Clavien-Dindo classification	471				0.587
0		225 (47.8%)	209 (47.4%)	16 (53.3%)	
I		12 (2.5%)	12 (2.7%)	0 (0.0%)	
II		90 (19.1%)	82 (18.6%)	8 (26.7%)	
III a		83 (17.6%)	81 (18.4%)	2 (6.7%)	
III b		30 (6.4%)	27 (6.1%)	3 (10.0%)	
IV a		21 (4.5%)	20 (4.5%)	1 (3.3%)	
IV b		7 (1.5%)	7 (1.6%)	0 (0.0%)	
V		3 (0.6%)	3 (0.7%)	0 (0.0%)	
*(Missing)*		*0*	*0*	*0*	
Renal thrombosis	471	3 (0.6%)	2 (0.5%)	1 (3.3%)	0.18
* (Missing)*		*0*	*0*	*0*	
Bleeding	471	29 (6.2%)	28 (6.3%)	1 (3.3%)	>0.9
(Missing)		0	0	0	
Lymphocele	471	45 (9.6%)	39 (8.8%)	6 (20.0%)	0.055
(Missing)		0	0	0	
Wound infection	471	23 (4.9%)	23 (5.2%)	0 (0.0%)	0.386
(Missing)		0	0	0	
Fascial dehiscence	471	8 (1.7%)	8 (1.8%)	0 (0.0%)	>0.9
(Missing)		0	0	0	
Myocardial infarction	471	18 (3.8%)	18 (4.1%)	0 (0.0%)	0.619
(Missing)		0	0	0	
Pulmonary embolism	471	9 (1.9%)	8 (1.8%)	1 (3.3%)	0.450
(Missing)		0	0	0	
Premature DJ-removal	54	13 (24.1%)	6 (25.0%)	7 (23.3%)	>0.9
(Missing)		417	417	0	
BKV positive^3^	471	59 (12.5%)	49 (11.1%)	10 (33.3%)	0.002
(Missing)		0	0	0	
BKV viraemia	471	46 (9.8%)	38 (8.6%)	8 (26.7%)	0.005
(Missing)		0	0	0	
BKV viruria	471	39 (8.3%)	31 (7.0%)	8 (26.7%)	0.002
(Missing)		0	0	0	
Cold ischemic time [min]	469	238 (174, 590)	238 (173, 578.5)	230.5 (182, 649)	0.757
(Missing)		2	2	0	
Warm ischemic time [min]	463	35 (30, 49)	35 (30, 49)	34 (29, 43.8)	0.302
* (Missing)*		*8*	*8*	*0*	
Recipient surgery duration [min]	468	154 (133, 200)	154 (133, 200)	158.5 (126.2, 186.2)	0.757
* (Missing)*		*3*	*3*	*0*	

¹n (%); Median (Q1, Q3).

²Fisher's exact test; Pearson's Chi-squared test; Wilcoxon rank sum test

³BK positive: BK positive was defined according to the predefined viral-load thresholds (>10^7^ copies/mL in urine or >10^4^ copies/mL in plasma)

Among patients with detectable BKV replication, the median urinary BKV load was 1.36 × 10^7^ copies/mL (IQR: 6.22 × 10^4^–1.00 × 10^8^). The median plasma BKV load was 3.75 × 10³ copies/mL (IQR 2.51 × 10²–2.72 × 10^4^). Clinically relevant BKV replication was observed significantly more frequently in the stenosis group. BKV viruria was present in 7.0% of the non-stenosis group compared to 26.7% in the stenosis group (p = 0.002), and BK viraemia was similarly more prevalent in the stenosis group (8.6% vs. 26.7%, p = 0.005).

### Survival data and kidney function

Patient survival did not differ significantly between the two groups (p = 0.833, **Figure 2**). In the non-stenosis group, patient survival was 94.6% at one year and 90.3% at three years, compared to 96.3% and 88.9% in the stenosis group, respectively. Overall graft survival was likewise comparable between groups (p = 0.2), with one- and three-year graft survival rates of 89.8% and 85.9% in the non-stenosis group, versus 90.0% and 76.7% in the stenosis group (**Figure 3**). Death-censored graft survival was significantly higher in the non-stenosis group (p = 0.01, **Figure 4**). One-year death-censored graft survival was 94.3% in the non-stenosis group and 90.0% in the stenosis group, with three-year rates of 93.3% and 79.7%, respectively.

**Figure 2:**
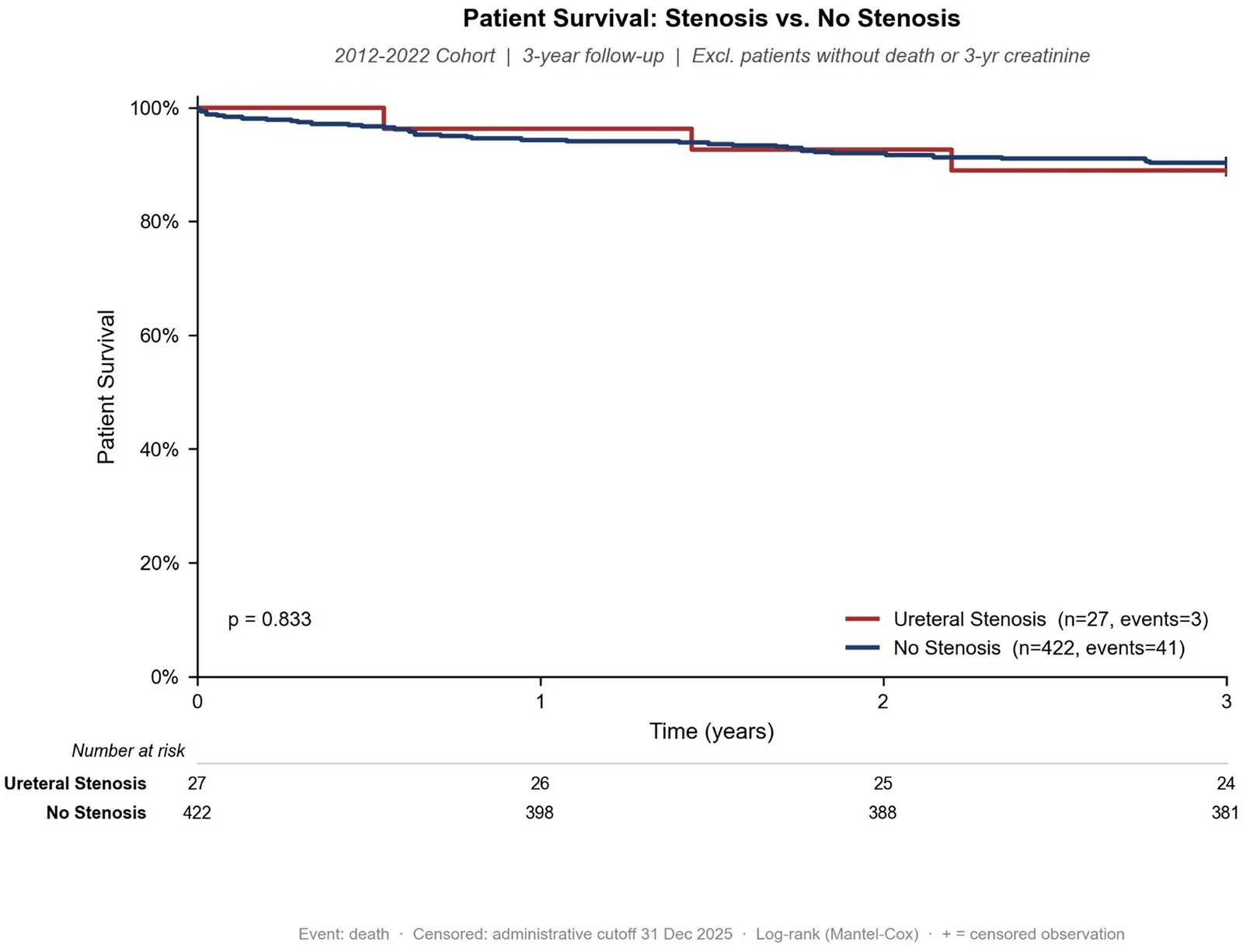
Kaplan-Meier curve depicting patient survival in the stenosis and non-stenosis groups over three years following kidney transplantation. No significant difference was observed between groups (p = 0.833, log-rank test). One-year survival was 96.3% vs. 94.6% and three-year survival 88.9% vs. 90.3% in the stenosis and non-stenosis groups, respectively.

**Figure 3:**
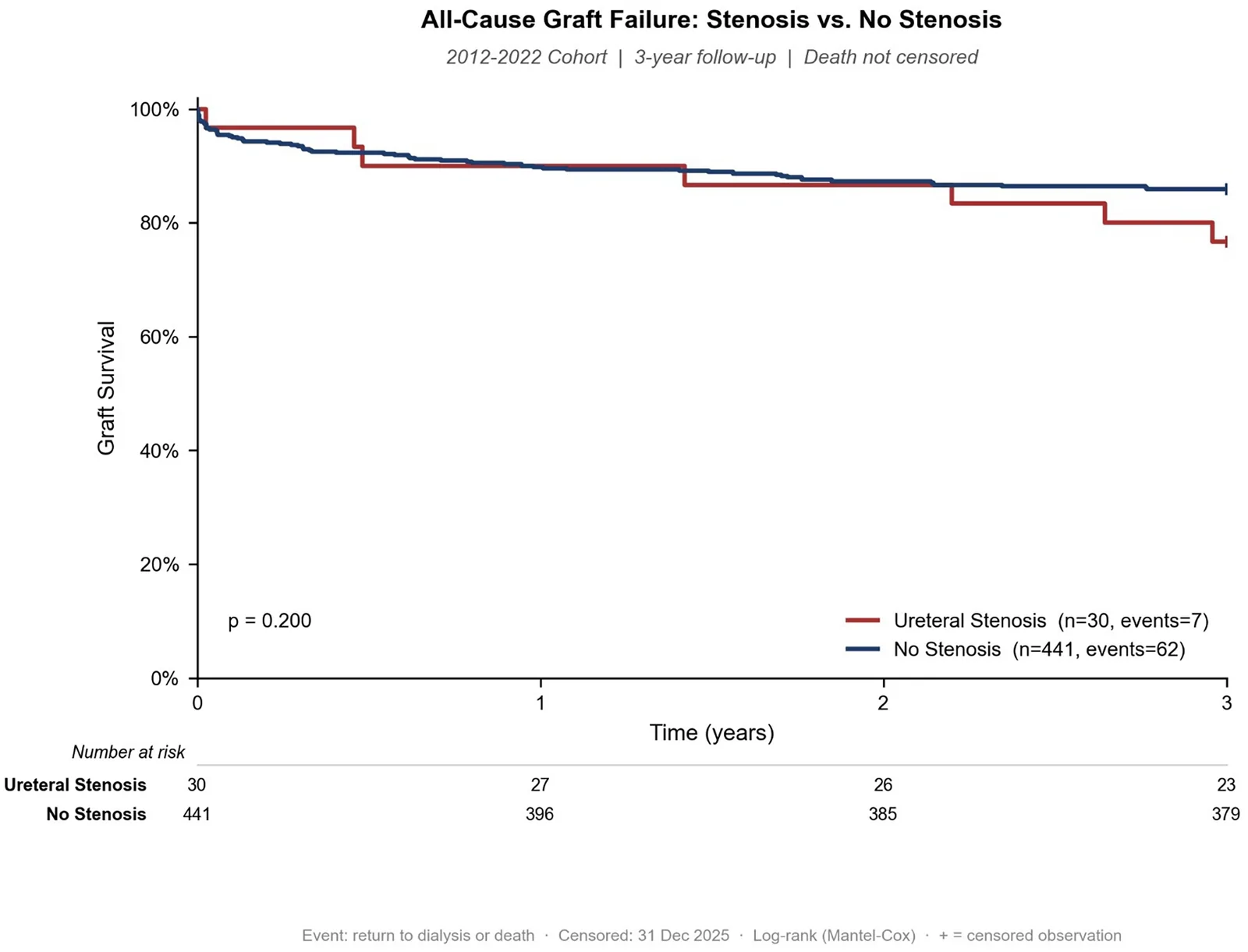
Kaplan-Meier curve depicting overall graft survival in the stenosis and non-stenosis groups over three years following kidney transplantation. No significant difference was observed between groups (p = 0.2, log-rank test). One- and three-year graft survival rates were 90.0% and 76.7% in the stenosis group versus 89.8% and 85.9% in the non-stenosis group, respectively.

**Figure 4:**
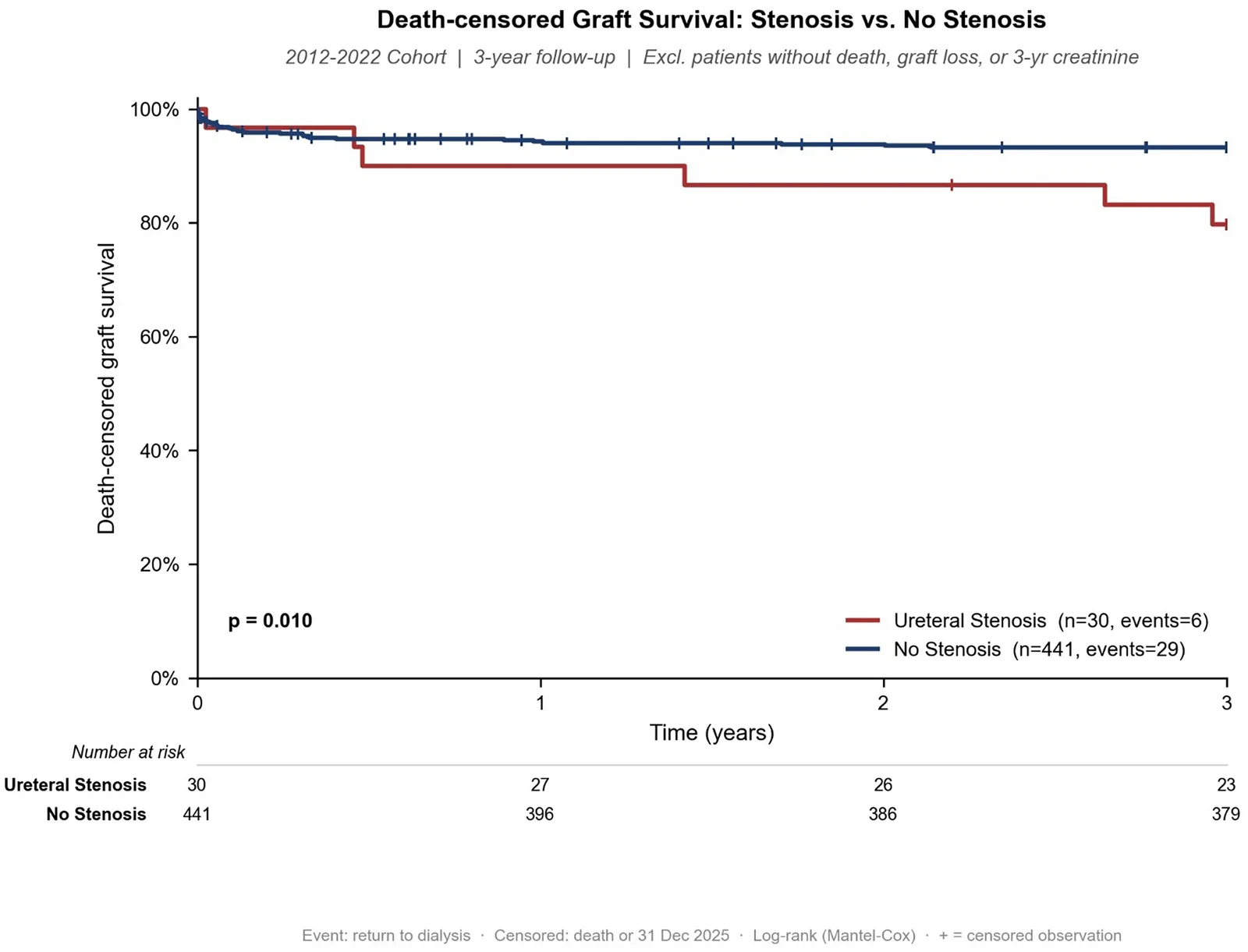
Kaplan-Meier curve depicting death-censored graft survival in the stenosis and non-stenosis groups over three years following kidney transplantation. Death-censored graft survival was significantly lower in the stenosis group (p = 0.01, log-rank test), with three-year rates of 79.7% versus 93.3% in the stenosis and non-stenosis groups, respectively.

Creatinine levels were consistently elevated in the stenosis group throughout routine follow-up (**Figure 5**). At discharge after transplantation, median creatinine levels were comparable between groups (1.660 mg/dL vs. 1.865 mg/dL, p = 0.809). Statistically significant differences emerged at six months (1.540 mg/dL vs. 1.890 mg/dL, p = 0.006), one year (1.5 mg/dL vs. 1.96 mg/dL, p = 0.004), and two years (1.5 mg/dL vs. 1.85 mg/dL, p = 0.017), with creatinine remaining persistently elevated in the stenosis group. By three years, the difference between groups narrowly failed to reach significance (1.5 mg/dL vs. 1.85 mg/dL, p = 0.079). Patients with ureteral stenosis were stratified into early and late stenosis groups based on time to diagnosis, defined as less than 90 days and 90 days or more, corresponding to the median time to diagnosis in the overall stenosis cohort (**Table 4**). Median time to diagnosis was 61 days in the early and 181 days in the late stenosis group (p < 0.001). Stenosis location was comparable between groups (p = 0.353): distal stenoses accounted for 86.7% and 66.7% of cases in the early and late groups, respectively, while proximal stenoses were identified in 13.3% and 25.0% of cases. Mid-ureteral stenosis was absent in the early group and present in one patient (8.3%) in the late group, with location data unavailable for the remaining patients in the late group. Median time between diagnosis of ureteral stenosis and surgical intervention was 34 days in the early group and 50.5 days in the late group (p = 0.308). Premature Double-J (DJ)-catheter removal, defined as removal prior to the scheduled 30-day interval was comparable between the early and late stenosis groups (26.7% vs. 20.0%, p > 0.9), as was the rate of DJ-catheter implantation following stenosis diagnosis (66.7% vs. 73.3%, p > 0.9).

**Figure 5:**
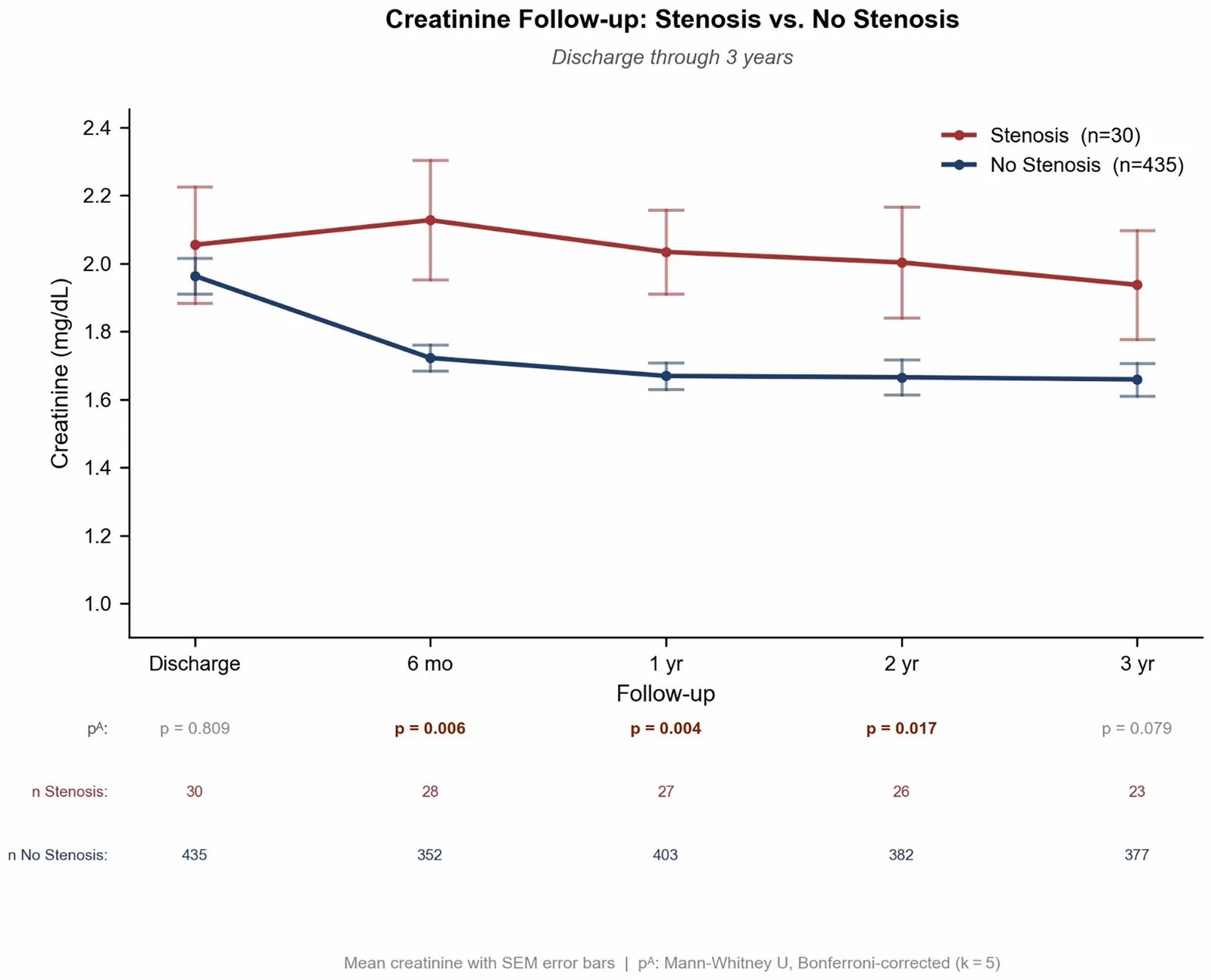
Longitudinal creatinine levels (mg/dL) in the stenosis and non-stenosis groups from transplantation discharge through three years of follow-up. Median creatinine was significantly elevated in the stenosis group at 6 months (1.54 vs. 1.89 mg/dL, p = 0.006), 1 year (1.50 vs. 1.96 mg/dL, p = 0.004), and 2 years (1.50 vs. 1.85 mg/dL, p = 0.017). Differences did not quite reach statistical significance at 3 years (p = 0.079). Data are presented as median with interquartile range.

**Table 4:** Ureteral stenosis stratified by incidence over time.

Characteristic	N	Overall	Early stenosis	Late stenosis	p-value²
		N = 30¹	N = 15¹	N = 15¹	
Days until ureter stenosis	*30*	*90 (61.2, 179.5)*	*61 (48, 77)*	*181 (128, 495.5)*	*<0.001*
(Missing)		*0*	*0*	*0*	
Ureter stenosis location	27				0.353
Distal		21 (77.8%)	13 (86.7%)	8 (66.7%)	
Medial		1 (3.7%)	0 (0.0%)	1 (8.3%)	
Proximal		5 (18.5%)	2 (13.3%)	3 (25.0%)	
(Missing)		*3*	*0*	*3*	
Days to surgery after stenosis	27	48 (20.5, 97)	34 (15, 76)	50.5 (32, 111.5)	0.308
(Missing)		*3*	*2*	*1*	
Premature DJ removal	30	7 (23.3%)	4 (26.7%)	3 (20.0%)	>0.9
(Missing)		*0*	*0*	*0*	
DJ after Ureter stenosis diagnosis	30	21 (70.0%)	10 (66.7%)	11 (73.3%)	>0.9
(Missing)		*0*	*0*	*0*	
Percutaneous nephrostomy	30	8 (26.7%)	3 (20.0%)	5 (33.3%)	0.682
(Missing)		*0*	*0*	*0*	
Rejection before ureter stenosis	27	3 (11.1%)	0 (0.0%)	3 (25.0%)	0.075
(Missing)		*3*	*0*	*3*	
Urinary tract infections before ureter stenosis	27	18 (66.7%)	11 (73.3%)	7 (58.3%)	0.448
(Missing)		*3*	*0*	*3*	
Creatinine at admission before revision [mg/dl]	27	2.0 (1.8, 2.7)	2.0 (1.8, 3.0)	1.9 (1.8, 2.6)	0.865
(Missing)		*3*	*2*	*1*	
Creatinine at discharge after revision [mg/dl]	26	1.8 (1.6, 2.2)	1.7 (1.5, 1.9)	1.9 (1.7, 2.5)	0.218
(Missing)		*4*	*2*	*2*	
Creatinine 6 months after revision [mg/dl]	23	1.9 (1.7, 2.3)	1.8 (1.7, 2.1)	2.3 (1.8, 3.1)	0.176
(Missing)		*7*	*3*	*4*	
Creatinine 1 year after revision [mg/dl]	24	1.8 (1.6, 2.4)	1.7 (1.5, 2.3)	2.0 (1.6, 3.2)	0.183
(Missing)		*6*	*4*	*2*	
Creatinine 2 years after revision [mg/dl]	23	1.7 (1.4, 2.0)	1.6 (1.4, 2.0)	1.8 (1.5, 3.7)	0.281
(Missing)		*7*	*4*	*3*	
Creatinine 3 years after revision [mg/dl]	20	1.7 (1.4, 2.1)	1.7 (1.4, 2.0)	1.7 (1.6, 2.2)	0.543
(Missing)		*10*	*4*	*6*	
Redialysis	30	6 (20.0%)	2 (13.3%)	4 (26.7%)	0.651
(Missing)		*0*	*0*	*0*	
Time of transplant until death [years]	8	3.7 (2.0, 4.9)	3.2 (2.0, 4.8)	3.9 (2.7, 4.9)	>0.9
(Missing)		*22*	*11*	*11*	
Death	28	8 (28.6%)	4 (28.6%)	4 (28.6%)	>0.9
(Missing)		*2*	*1*	*1*	

¹Median (Q1, Q3); n (%).

²Wilcoxon rank sum test; Fisher's exact test

### Revision of ureter stenosis

Revision therapy data were available for 29 patients (**Table 5**), of whom 17 underwent transposition and 12 ureteroneocystostomy. The two revision groups were comparable regarding age, donor type, sex, preexisting diseases, urinary tract infection history, premature DJ-catheter removal, and rejection episodes preceding ureteral stenosis. Time from stenosis diagnosis to surgical intervention, operative duration, intensive care unit (ICU) stay, postoperative DJ-catheter use, and length of hospital stay were all comparable between revision strategies, though operative duration (105 vs. 85 min, p = 0.078) and time from diagnosis to intervention (35 vs 58 days, p = 0.066) showed numerical trends towards longer values in the transposition group without reaching statistical significance. One patient with ureteral stenosis did not undergo surgical revision and was managed conservatively with long term stenting. Creatinine levels measured prior to revision and at discharge, six months, one, two, and three years postoperatively showed no significant differences between the two revision approaches. Graft failure occurred in 3 of 17 (17.6%) transposition and 1 of 12 (8.3%) ureteroneocystostomy patients, without significant difference (p = 0.622) (**Figure 6**).

**Table 5:** Ureteral stenosis: transposition vs ureteroneocystostomy.

Characteristic	N	Overall	Transposition	Ureteroneocystostomy	p-value²
		N = 29¹	N = 17¹	N = 12¹	
Recipient age at surgery [years]	29	50 (33, 65)	50 (29, 66)	49.5 (36.2, 63.5)	0.674
*(Missing)*		*0*	*0*	*0*	
Recipient sex	29				0.119
Female		11 (37.9%)	4 (23.5%)	7 (58.3%)	
Male		18 (62.1%)	13 (76.5%)	5 (41.7%)	
*(Missing)*		*0*	*0*	*0*	
Living Donation	29	16 (55.2%)	9 (52.9%)	7 (58.3%)	>0.9
*(Missing)*		*0*	*0*	*0*	
Disease group	29				0.078
Atrophic Kidneys		0 (0.0%)	0 (0.0%)	0 (0.0%)	
Congenital/Hereditary Kidney Diseases		2 (6.9%)	2 (11.8%)	0 (0.0%)	
Cystic Kidney Diseases		9 (31.0%)	2 (11.8%)	7 (58.3%)	
Diabetic Nephropathy		2 (6.9%)	2 (11.8%)	0 (0.0%)	
Glomerular Diseases		9 (31.0%)	5 (29.4%)	4 (33.3%)	
Hypertensive/Vascular Nephropathy		3 (10.3%)	3 (17.6%)	0 (0.0%)	
Infectious/post-infectious		0 (0.0%)	0 (0.0%)	0 (0.0%)	
Neoplastic		0 (0.0%)	0 (0.0%)	0 (0.0%)	
Others		0 (0.0%)	0 (0.0%)	0 (0.0%)	
Systemic Diseases Affecting Kidneys		2 (6.9%)	1 (5.9%)	1 (8.3%)	
Transplant-Related		0 (0.0%)	0 (0.0%)	0 (0.0%)	
Traumatic/Iatrogenic		0 (0.0%)	0 (0.0%)	0 (0.0%)	
Tubulointerstitial Diseases		0 (0.0%)	0 (0.0%)	0 (0.0%)	
Unknown		2 (6.9%)	2 (11.8%)	0 (0.0%)	
*(Missing)*		*0*	*0*	*0*	
Hypertension	29	23 (79.3%)	15 (88.2%)	8 (66.7%)	0.198
*(Missing)*		*0*	*0*	*0*	
Diabetes mellitus	29	3 (10.3%)	3 (17.6%)	0 (0.0%)	0.246
*(Missing)*		*0*	*0*	*0*	
Coronary heart disease	29	1 (3.4%)	0 (0.0%)	1 (8.3%)	0.414
*(Missing)*		*0*	*0*	*0*	
Peripheral artery disease	29	1 (3.4%)	0 (0.0%)	1 (8.3%)	0.414
*(Missing)*		*0*	*0*	*0*	
Days from transplant to ureter stenosis	29	86 (60, 181)	71 (58, 175)	96 (75, 187)	0.58
*(Missing)*		*0*	*0*	*0*	
Late stenosis (≥90 days)	29	13 (44.8%)	7 (41.2%)	6 (50.0%)	>0.9
*(Missing)*		*0*	*0*	*0*	
Stenosis location	27				0.199
Distal		23 (85.2%)	12 (75.0%)	11 (100.0%)	
Medial		1 (3.7%)	1 (6.2%)	0 (0.0%)	
Proximal		3 (11.1%)	3 (18.8%)	0 (0.0%)	
*(Missing)*		*2*	*1*	*1*	
Premature DJ removal	29	6 (20.7%)	2 (11.8%)	4 (33.3%)	0.198
*(Missing)*		*0*	*0*	*0*	
DJ after ureter stenosis diagnosis	29	22 (75.9%)	11 (64.7%)	11 (91.7%)	0.187
*(Missing)*		*0*	*0*	*0*	
Percutaneous nephrostomy	29	7 (24.1%)	6 (35.3%)	1 (8.3%)	0.187
*(Missing)*		*0*	*0*	*0*	
Rejection before ureter stenosis	28	3 (10.7%)	1 (5.9%)	2 (18.2%)	0.543
*(Missing)*		*1*	*0*	*1*	
BK before ureter stenosis	29	10 (34.5%)	6 (35.3%)	4 (33.3%)	>0.9
*(Missing)*		*0*	*0*	*0*	
Urinary tract infection before ureter stenosis	28	14 (50.0%)	8 (47.1%)	6 (54.5%)	>0.9
*(Missing)*		*1*	*0*	*1*	
Initial reconstruction of ureter	29				
Lich-Gregoir		0 (0.0%)	0 (0.0%)	0 (0.0%)	
Politano-Leadbetter		29 (100.0%)	17 (100.0%)	12 (100.0%)	
Other		0 (0.0%)	0 (0.0%)	0 (0.0%)	
*(Missing)*		*0*	*0*	*0*	
Days to surgery after stenosis diagnosis	29	51 (21, 77)	35 (15, 62)	58 (46.2, 119.8)	0.066
*(Missing)*		*0*	*0*	*0*	
Surgery duration [min]	28	93.5 (81.8, 114.2)	105 (90, 118)	85 (70, 99)	0.078
*(Missing)*		*1*	*0*	*1*	
Blood loss [ml]	10	200 (100, 200)	200 (150, 250)	100 (75, 150)	0.19
*(Missing)*		*19*	*10*	*9*	
Hospital stay after revision [days]	28	8 (8, 14.2)	8 (8, 10)	9 (8, 17)	0.423
*(Missing)*		*1*	*0*	*1*	
Creatinine at admission before revision [mg/dl]	29	1.9 (1.7, 2.7)	1.9 (1.8, 2.7)	1.8 (1.6, 2.5)	0.479
*(Missing)*		*0*	*0*	*0*	
Creatinine at discharge after revision [mg/dl]	28	1.9 (1.6, 2.5)	1.9 (1.7, 2.2)	1.9 (1.4, 2.7)	>0.9
*(Missing)*		*1*	*0*	*1*	
Creatinine 6 months after revision [mg/dl]	25	1.8 (1.5, 2.3)	2.0 (1.8, 2.3)	1.7 (1.5, 1.8)	0.233
*(Missing)*		*4*	*2*	*2*	
Creatinine 1 year after revision [mg/dl]	26	1.7 (1.6, 2.3)	1.7 (1.6, 2.3)	1.7 (1.5, 2.2)	0.775
*(Missing)*		*3*	*2*	*1*	
Creatinine 2 years after revision [mg/dl]	26	1.7 (1.4, 2.0)	1.8 (1.5, 2.0)	1.6 (1.4, 2.0)	0.856
*(Missing)*		*3*	*2*	*1*	
Creatinine 3 years after revision [mg/dl]	21	1.7 (1.4, 2.2)	1.9 (1.6, 2.7)	1.5 (1.4, 1.7)	0.177
*(Missing)*		*8*	*5*	*3*	
Graft failure	29	4 (13.8%)	3 (17.6%)	1 (8.3%)	0.622
*(Missing)*		*0*	*0*	*0*	
Time from transplant to redialysis [days]	4	571 (173.8, 994.8)	966 (566.5, 1,023.5)	176 (176, 176)	
*(Missing)*		*25*	*14*	*11*	
Time from transplant until death [years]	1	0.6 (0.6, 0.6)	NA (NA, NA)	0.6 (0.6, 0.6)	
*(Missing)*		*28*	*17*	*11*	
Death	29	1 (3.4%)	0 (0.0%)	1 (8.3%)	0.414
*(Missing)*		*0*	*0*	*0*	

¹Median (Q1, Q3); n (%).

²Wilcoxon rank sum test; Fisher's exact test; Pearson's Chi-squared test

**Figure 6:**
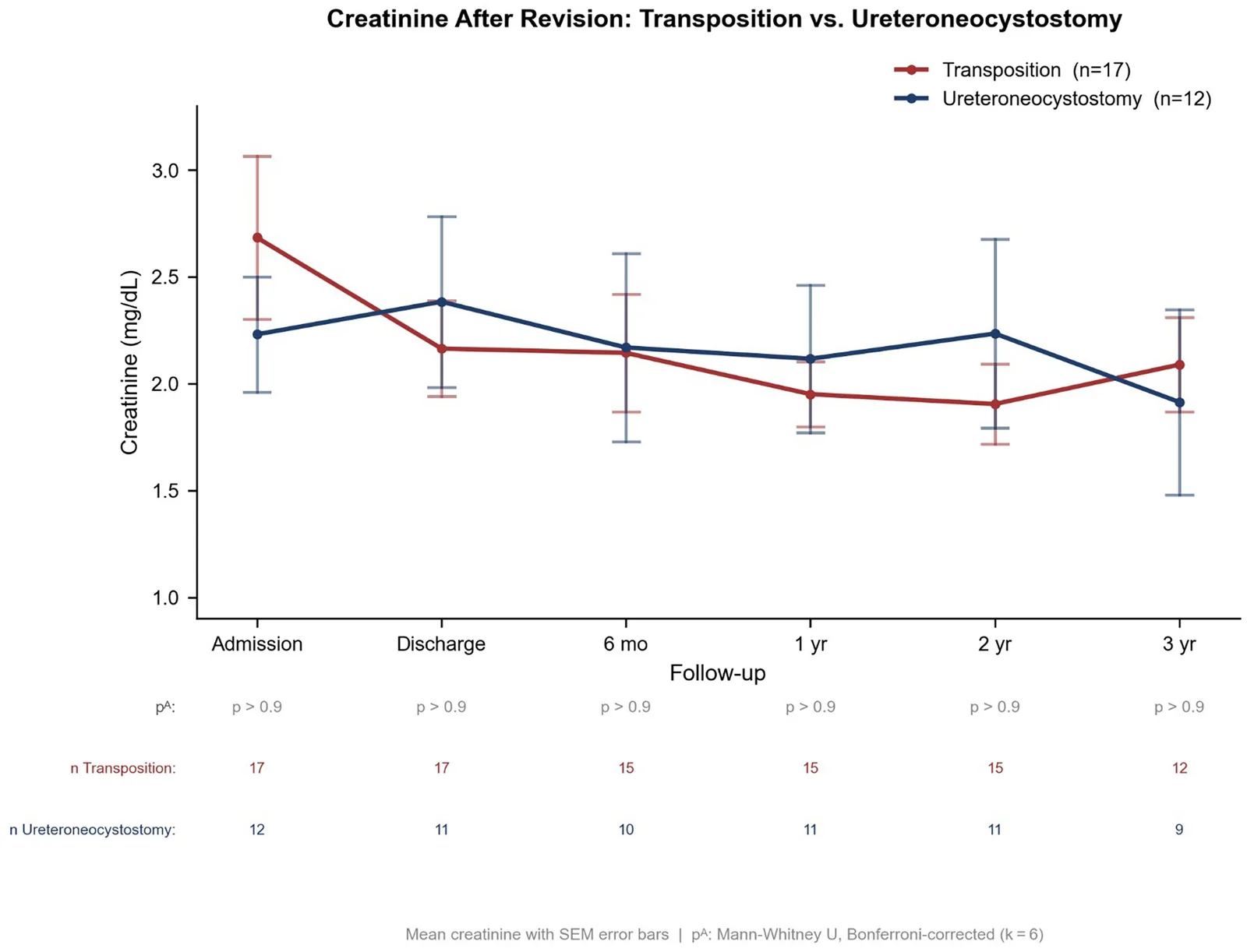
Longitudinal creatinine levels (mg/dL) in patients undergoing revision surgery, comparing transposition (n = 17) and ureteroneocystostomy (n = 12) from pre-revision through three years of follow-up. No significant differences in creatinine levels were observed between the two revision groups at any postoperative time point. Data are presented as median with interquartile range.

## Discussion

The present study reports a ureteral stenosis incidence of 6.37% following renal transplantation, which compares favorably with the broad range of 0.5–20% reported in the existing literature (9, 18, 20). The majority of stenoses were distally located and occurred within the first post-transplant year. The true incidence of ureteral stenosis may be underestimated: ultrasound findings of hydronephrosis, particularly when mild, can be difficult to interpret (18, 38). Pelvicalyceal dilatation of the denervated graft may represent a normal post-transplant finding rather than a sign of true obstruction, creating a diagnostic dilemma that may result in asymptomatic or subclinical strictures remaining undetected and intervention being delayed (39, 40).

The temporal distribution of stenosis onset, with early cases occurring at a median of 61 days and late cases at 181 days is consistent with the biphasic etiology of post-transplant ureteral stenosis, in which early strictures predominantly reflect ischemic injury, surgical factors, and periureteral fibrosis, whereas late-onset disease is more commonly attributed to viral-mediated urothelial damage, particularly BK virus (9). To further characterize the biphasic etiology of post-transplant ureteral stenosis, early and late stenosis subgroups were compared with respect to donor-related risk factors (**Supplementary Tables S1**–**3**). In accordance with German transplantation law, all deceased donor grafts in this cohort were procured following brain death determination (DBD); donation after circulatory death (DCD) is not legally permitted in Germany (41) thereby eliminating DCD-associated warm ischemic injury as a confounding variable. According to the UNOS definition, ECD included deceased donors aged ≥60 years, or aged 50–59 years with at least two of the following: (i) cerebrovascular cause of death, (ii) history of hypertension, and/or (iii) terminal serum creatinine >1.5 mg/dL (42). Notably, among deceased donors, all early stenosis cases involved donors aged ≥60 years, fulfilling a primary ECD criterion, compared to 50.0% in the late stenosis group (p = 0.085), a finding consistent with prior evidence that donor age and ischemic susceptibility act synergistically in promoting ureteral injury (19, 43). The proportion of grafts with multiple renal arteries did not differ significantly between groups (42.9% vs. 37.5%, p > 0.9). In contrast, BK viruria and viraemia were numerically more prevalent in the late stenosis group (50.0% vs. 20.0% and 42.9% vs. 13.3%, respectively), consistent with a viral-mediated mechanism predominating in late-onset disease, although these differences did not reach statistical significance, likely due to the limited sample size (**Supplementary Table S1**).

The cohort was well-balanced with respect to recipient characteristics, including age, sex, BMI, and implantation side, supporting the comparability of the two groups. Older donor age was significantly associated with ureteral stenosis (65 vs. 56 years, p = 0.006), consistent with prior reports implicating impaired ureteral perfusion and reduced healing capacity in older grafts as predisposing factors for fibrotic stricture formation (9). The presence of multiple renal arteries was likewise strongly associated with stenosis development (p < 0.001), echoing previous studies linking complex vascular anatomy with compromised ureteral blood supply and subsequent ischemic injury (5, 8, 19).

Extrinsic factors also warrant consideration: periureteral lymphoceles may cause ureteral compression and present clinically similar to intrinsic strictures. Postoperative hematomas may exert external pressure on the ureter, promoting secondary ischemia, and contributing to stenosis formation (26, 44). Cases in which ureteral obstruction was attributable exclusively to lymphoceles and resolved following their treatment were excluded from the present analysis (44).The perioperative course was comparable between groups, with no significant differences in ischemic times, delayed graft function, primary non-function (PNF), or postoperative surgical complications. Lymphocele incidence was numerically higher in the stenosis group (8.8% vs. 20.0%), though this did not reach statistical significance (p = 0.055) calling for further investigations in larger cohorts. Of particular interest was the significantly elevated rate of BK viruria (7.0% vs. 26.7%, p = 0.002) and BK viraemia (p = 0.005) in the stenosis group. Mechanistically, BK virus is well established as a cause of ureteral inflammation and epithelial damage, and its association with ureteral stenosis in the present cohort is consistent with prior literature implicating viral-mediated urothelial injury in stricture pathogenesis (15, 16). As the viral screening protocols evolved technically over the extended study period, its diagnostic sensitivity was affected. This limitation in diagnostic sensitivity, as compared to current protocols, may have led to an underestimation of BK virus incidence, particularly in earlier cases (14). Nevertheless, the significantly higher frequency of detectable BKV replication in the stenosis group reinforces the clinical relevance of BK virus monitoring in post-transplant surveillance.

Patient survival and overall graft survival did not differ significantly between groups, which may partly reflect the effectiveness of timely surgical revision and the relatively small sample size of the stenosis group. However, death-censored graft survival was significantly lower in the stenosis group at three years (79.7% vs. 93.3%, p = 0.01), highlighting the long-term impact of ureteral stenosis on graft integrity independent of patient mortality (**Figure 4**). This finding is corroborated by the persistently impaired kidney transplant function observed in the stenosis group from six months onwards. Notably, creatinine differences were no longer statistically significant at three years (p = 0.079), which may be due to loss of follow up and restricted sample size.

Among the available techniques, the extravesical Lich–Gregoir approach is preferred due to its avoidance of cystotomy, technical simplicity, reduced operative time, and suitability for shorter ureters (13, 21, 23). The rich vascular supply of the bladder additionally supports optimal healing of the ureterovesical anastomosis (24, 25). For proximal or mid-ureteral lesions, pyeloureterostomy and ureteroureterostomy represent viable alternatives, provided the integrity of the native ureter is confirmed (26). In the present cohort, both transposition and ureteroneocystostomy yielded comparable functional outcomes, with no significant differences in creatinine levels at any postoperative time point. This supports the use of either approach as a therapeutic option, with the choice guided by individual patient anatomy, stenosis characteristics, and surgeon expertise. Direct comparisons between these surgical techniques remain limited in the literature, as most studies focus on comparisons between open and endoscopic approaches (10). The observation that revision surgery was exclusively required following Politano-Leadbetter reconstruction may reflect a historical institutional preference for this technique during the early study period rather than an inherent inferiority of the method, as contemporary practice at our center has shifted toward Lich-Gregoir anastomosis in line with current guidelines (13, 22).

The limitations of this study include its retrospective, single-center design, which may limit generalizability. The evolving viral screening protocols over the extended study period may have introduced diagnostic variability, particularly with respect to BK virus detection. Additionally, the relatively small size of the stenosis subgroup limits the statistical power of certain subgroup analyses. A statistically significant association between rejection history and proximal or medial stenosis location was observed (p < 0.001, **Supplementary Table S1**), though the extremely small subgroup sizes (n=1 medial, n=4 proximal) preclude reliable interpretation of this finding. Prospective multicenter studies are needed to validate these findings.

Occurring in 6.37% of kidney transplant recipients, ureteral stenosis was significantly associated with extended criteria donor characteristics, particularly older donor age, multiple renal arteries, and BK virus infection. Although overall patient and graft survival were unaffected, death-censored graft survival was significantly reduced and kidney transplant function impaired. This highlights the need for early detection and timely intervention. Surgical revision effectively stabilized renal function regardless of technique employed. Among revision cases, no statistically significant differences in long-term renal function or graft-survival were observed between the two revision groups. However, given the small subgroup sizes, these findings should not be interpreted as evidence of equivalence. Prospective multicenter studies are warranted to validate these findings and further optimize surveillance and surgical strategies in the post-transplant setting.

## Data Availability

The original contributions presented in the study are included in the article/**Supplementary Material**. Further inquiries can be directed to the corresponding author.

## Supplementary material

Supplementary Figure 1 Surgical revision techniques for ureteral complications after kidney transplantation. The applied surgical techniques were ureteroneocystostomy, ureteroureterostomy and pyeloureterostomy. Ureteroureterostomy and pyeloureterostomy, both involving anastomosis to the native ureter, were grouped together as “transposition” and compared with ureteroneocystostomy. The surgical techniques are illustrated schematically.
