# Supplementary material for: Post-transplant ureteral stenosis: donor-related risk factors, graft outcome and surgical management
Source: Front Nephrol. 2026 Sep 24;6:1943229. doi: 10.3389/fneph.2026.1943229 (PMC13645714; doi:10.3389/fneph.2026.1943229)
Supplement: Supplementary file 1 [file Table1.docx]

| **Table S1:** |  |  |  |  |  |  |
| --- | --- | --- | --- | --- | --- | --- |
| **Ureteral Stenosis Stratified by Location** |  |  |  |  |  |  |
| **Characteristic** | **N** | **Overall** | **Distal stenosis** | **Medial stenosis** | **Proximal stenosis** | **p-value²** |
|  |  | **N = 27¹** | **N = 21¹** | **N = 1¹** | **N = 5¹** |  |
| Late stenosis | 27 | 12 (44.4%) | 8 (38.1%) | 1 (100.0%) | 3 (60.0%) | 0.353 |
| *(Missing)* |  | *0* | *0* | *0* | *0* |  |
| Days after transplant until ureter stenosis | 27 | 86 (57.5, 178) | 79 (54, 165) | 698 (698, 698) | 95 (88, 351) | 0.298 |
| *(Missing)* |  | *0* | *0* | *0* | *0* |  |
| Days to surgery after ureter stenosis | 24 | 50.5 (20.8, 86.5) | 48 (20.5, 97) | 35 (35, 35) | 62 (43.5, 118.5) | 0.626 |
| *(Missing)* |  | *3* | *2* | *0* | *1* |  |
| Premature DJ removal | 27 | 7 (25.9%) | 6 (28.6%) | 0 (0.0%) | 1 (20.0%) | 0.772 |
| *(Missing)* |  | *0* | *0* | *0* | *0* |  |
| DJ after ureter stenosis diagnosis | 27 | 19 (70.4%) | 15 (71.4%) | 1 (100.0%) | 3 (60.0%) | 0.708 |
| *(Missing)* |  | *0* | *0* | *0* | *0* |  |
| Percutaneous nephrostomy | 27 | 7 (25.9%) | 5 (23.8%) | 1 (100.0%) | 1 (20.0%) | 0.223 |
| *(Missing)* |  | *0* | *0* | *0* | *0* |  |
| *Rejection before ureter stenosis* | *25* | *2 (8.0%)* | *0 (0.0%)* | *1 (100.0%)* | *1 (25.0%)* | ***<0.001*** |
| *(Missing)* |  | *2* | *1* | *0* | *1* |  |
| BK before ureter stenosis | 10 | 9 (90.0%) | 7 (100.0%) | 0 (NA%) | 2 (66.7%) | 0.645 |
| *(Missing)* |  | *17* | *14* | *1* | *2* |  |
| CMV before ureter stenosis | 4 | 1 (25.0%) | 1 (33.3%) | 0 (NA%) | 0 (0.0%) | >0.9 |
| *(Missing)* |  | *23* | *18* | *1* | *4* |  |
| Urinary tract infection before ureter stenosis | 25 | 16 (64.0%) | 13 (65.0%) | 1 (100.0%) | 2 (50.0%) | 0.634 |
| *(Missing)* |  | *2* | *1* | *0* | *1* |  |
| Creatinine at admission before revision [mg/dl] | 24 | 1.9 (1.8, 2.7) | 1.9 (1.7, 2.7) | 6.0 (6.0, 6.0) | 1.9 (1.8, 2.0) | 0.715 |
| *(Missing)* |  | *3* | *2* | *0* | *1* |  |
| Creatinine at discharge after revision [mg/dl] | 23 | 1.7 (1.5, 2.1) | 1.8 (1.5, 2.2) | 5.2 (5.2, 5.2) | 1.7 (1.6, 1.8) | 0.67 |
| *(Missing)* |  | *4* | *3* | *0* | *1* |  |
| Creatinine 6 months after revision [mg/dl] | 21 | 1.8 (1.7, 2.3) | 1.8 (1.7, 2.3) | 5.8 (5.8, 5.8) | 1.9 (1.8, 2.3) | 0.508 |
| *(Missing)* |  | *6* | *5* | *0* | *1* |  |
| Creatinine 1 year after revision [mg/dl] | 21 | 1.7 (1.5, 2.4) | 1.7 (1.4, 2.4) | 13.5 (13.5, 13.5) | 1.8 (1.7, 2.1) | 0.791 |
| *(Missing)* |  | *6* | *4* | *0* | *2* |  |
| Creatinine 2 years after revision [mg/dl] | 20 | 1.7 (1.4, 1.9) | 1.6 (1.4, 1.9) | 13.2 (13.2, 13.2) | 1.7 (1.6, 1.9) | 0.737 |
| *(Missing)* |  | *7* | *5* | *0* | *2* |  |
| Creatinine 3 years after revision [mg/dl] | 18 | 1.7 (1.4, 2.0) | 1.7 (1.4, 1.9) | 11.1 (11.1, 11.1) | 1.7 (1.6, 2.2) | 0.449 |
| *(Missing)* |  | *9* | *7* | *0* | *2* |  |
| Redialysis | 27 | 5 (18.5%) | 3 (14.3%) | 1 (100.0%) | 1 (20.0%) | 0.097 |
| *(Missing)* |  | *0* | *0* | *0* | *0* |  |
| Death | 25 | 7 (28.0%) | 4 (20.0%) | 0 (NA%) | 3 (60.0%) | 0.221 |
| *(Missing)* |  | *2* | *1* | *1* | *0* |  |
|  |  |  |  |  |  |  |
| *¹ n (%); Median (Q1, Q3)* |  |  |  |  |  |  |
| *² Fisher's exact test; Kruskal-Wallis rank sum test* |  |  |  |  |  |  |

| **Table S2:** |  |  |  |  |  |
| --- | --- | --- | --- | --- | --- |
| **Extended Criteria Donor (ECD)-associated Characteristics in Deceased Donors, Stratified by Stenosis Onset** | | |  |  |  |
| **Criterion** | **N** | **Overall** | **Early stenosis** | **Late stenosis** | **p-value** |
|  |  | **N = 15** | **N = 7** | **N = 8** |  |
| **Donor age >=60 years** | 14 | 10 (71.4%) | 6 (100.0%) | 4 (50.0%) | 0.085 |
| *(Missing)* |  | *1* | *1* | *0* |  |
| **Multiple arteries (>1)** | 15 | 6 (40.0%) | 3 (42.9%) | 3 (37.5%) | >0.9 |
| *(Missing)* |  | *0* | *0* | *0* |  |
| **Total ischemia time >24 hours** | 15 | 0 (0.0%) | 0 (0.0%) | 0 (0.0%) |  |
| *(Missing)* |  | *0* | *0* | *0* |  |
| **Donor age 50-59 years** | 14 | 1 (7.1%) | 0 (0.0%) | 1 (12.5%) | >0.9 |
| *(Missing)* |  | *1* | *1* | *0* |  |
| With diabetes | 0 | 0 (NA%) | 0 (NA%) | 0 (NA%) |  |
| With hypertension | 0 | 0 (NA%) | 0 (NA%) | 0 (NA%) |  |
| With creatinine >1.5 mg/dL | 0 | 0 (NA%) | 0 (NA%) | 0 (NA%) |  |
| With >=2 of the above | 0 | 0 (NA%) | 0 (NA%) | 0 (NA%) |  |
| **Donor age <50 years** | 14 | 3 (21.4%) | 0 (0.0%) | 3 (37.5%) | 0.209 |
| *(Missing)* |  | *1* | *1* | *0* |  |
| With diabetes | 0 | 0 (NA%) | 0 (NA%) | 0 (NA%) |  |
| With hypertension | 0 | 0 (NA%) | 0 (NA%) | 0 (NA%) |  |
| With creatinine >1.5 mg/dL | 0 | 0 (NA%) | 0 (NA%) | 0 (NA%) |  |
| With >=2 of the above | 0 | 0 (NA%) | 0 (NA%) | 0 (NA%) |  |
|  |  |  |  |  |  |
| *n (%)* |  |  |  |  |  |
| *Fisher's exact test* |  |  |  |  |  |
| *Hypertension: systolic blood pressure >139 mmHg or antihypertensive medication.* | |  |  |  |  |
| *Creatinine: donor creatinine before surgery (d_crea_before_surgery).* |  |  |  |  |  |
| *Total ischemia time >24 h based on cold ischemic time (>1440 min).* |  |  |  |  |  |
| *Sub-rows: n (%) within the respective age subgroup.* |  |  |  |  |  |

| **Table S3:** |  |  |  |  |  |
| --- | --- | --- | --- | --- | --- |
| **Extended Criteria Donor (ECD)-analogous characteristics in living donors, stratified by stenosis onset** | | |  |  |  |
| **Criteria** | **N** | **Overall** | **Early stenosis** | **Late stenosis** | **p-value** |
|  |  | **N = 15** | **N = 8** | **N = 7** |  |
| **Donor age >=60 years** | 15 | 9 (60.0%) | 4 (50.0%) | 5 (71.4%) | 0.608 |
| *(Missing)* |  | *0* | *0* | *0* |  |
| **Multiple arteries (>1)** | 15 | 4 (26.7%) | 1 (12.5%) | 3 (42.9%) | 0.282 |
| *(Missing)* |  | *0* | *0* | *0* |  |
| **Donor age 50-59 years** | 15 | 5 (33.3%) | 3 (37.5%) | 2 (28.6%) | >0.9 |
| *(Missing)* |  | *0* | *0* | *0* |  |
| With diabetes | 5 | 0 (0.0%) | 0 (0.0%) | 0 (0.0%) |  |
| With hypertension | 5 | 2 (40.0%) | 2 (66.7%) | 0 (0.0%) | 0.4 |
| With >=2 of the above | 5 | 0 (0.0%) | 0 (0.0%) | 0 (0.0%) |  |
| **Donor age <50 years** | 15 | 1 (6.7%) | 1 (12.5%) | 0 (0.0%) | >0.9 |
| *(Missing)* |  | *0* | *0* | *0* |  |
| With diabetes | 1 | 0 (0.0%) | 0 (0.0%) | 0 (NA%) |  |
| With hypertension | 1 | 0 (0.0%) | 0 (0.0%) | 0 (NA%) |  |
| With >=2 of the above | 1 | 0 (0.0%) | 0 (0.0%) | 0 (NA%) |  |
|  |  |  |  |  |  |
| *n (%)* |  |  |  |  |  |
| *Fisher's exact test* |  |  |  |  |  |
| *Hypertension: systolic blood pressure >139 mmHg or antihypertensive medication.* | |  |  |  |  |
| *Total ischemia time >24 h based on cold ischemic time (>1440 min).* |  |  |  |  |  |
| *Connective tissue disease criterion omitted: no donor-level variable available in dataset.* | |  |  |  |  |
| *Sub-rows: n (%) within the respective age subgroup.* |  |  |  |  |  |
